# Comparison of the sensitivity of coprological methods in detecting *Anoplocephala perfoliata* invasions

**DOI:** 10.1007/s00436-014-3919-4

**Published:** 2014-04-29

**Authors:** Krzysztof Tomczuk, Krzysztof Kostro, Klaudiusz Oktawian Szczepaniak, Maciej Grzybek, Maria Studzińska, Marta Demkowska-Kutrzepa, Monika Roczeń-Karczmarz

**Affiliations:** University of Life Sciences in Lublin, Lublin, Poland

**Keywords:** *Anoplocephala perfoliata*, Gravid proglottid, Coprological methods, Sensitivity

## Abstract

The autopsy of 487 slaughter horses revealed the presence of *Anoplocephala perfoliata* in 36 animals. The invasions varied in the intensity (3 to 2,069 tapeworms) and in the level of tapeworms’ proglottid maturity. Twenty nine horses were found to contain tapeworms with gravid proglottid. Fecal samples collected from the rectum were tested using following techniques: flotation with solution-saturated NaCl, decantation, McMaster’s, and modified sedimentation-flotation methods (50 g feces samples, flotation solution-saturated NaCl and sucrose, specific gravity 1.25 g/ml). The number of *A. perfoliata* positive fecal samples was significantly higher using the sedimentation-flotation methods 21 (58.33 %) than flotation 6 (16.66 %), decantation 3 (8.33 %), and McMaster’s 1 (2.77 %) techniques. The sensitivities of the coprological methods during the patent period were 20.69, 10.34, 3.45, and 72.41 % for the flotation, decantation, McMaster’s, and sedimentation-flotation method, respectively. Sedimentation–flotation techniques proved to be more sensitive than other one. The lowest intensity of invasion possible to detect using this method was nine tapeworms with gravid proglottid.

## Introduction

For many years, equine anoplocephalosis has been considered to be incidental finding in the intestinal tracts of horses at post mortem examination and rarely associated with clinical disease. However, nowadays, it is an emerging problem for both breeders and veterinary surgeons (Gundlach et al. 2004; Kornaś et al. 2007, 2010; Rehbein et al. 2013). *Anoplocephala perfoliata* belongs to the family of *Anoplocephalidae* (*Cyclophyllidea*, *Cestoda*) and has an indirect life cycle via pasture dwelling oribatid mites from *Oribatidae* spp. The tapeworm infestation in equines results from pasture invasion. Mites containing infective cysticercoids of the parasite are ingested by grazing horses. The parasite attaches to the intestinal mucosa of the ileocaecal junction with the suckers on the scolex. It matures to an adult in 6–10 weeks and attains the size of only 5–8 cm in length. Adult parasites shed gravid proglottids that break up during passage through the horse’s large intestine (Deplazes et al. 2013; Schnieder 2006).

Several authors have widely described pathology of this invasion including type of distribution (clustered or dispersed) and pathology. Mucosal ulceration, submucosal oedema, hypertrophy of the distal ileum, and decreased ileocaecal valve distensibility have all been reported to occur at the site of parasite attachment, and the severity of pathology is directly proportional to parasite infection intensity (Pavone et al. 2011; De Almeida et al. 2008). The mechanisms by which this pathology arises remain unexplored, but it would seem reasonable to consider both mechanical obstruction and parasite-derived antigens in these processes. The infected animals usually demonstrate a good nutrition status although several clinical conditions have been associated with *A. perfoliata* infections in horses (Deplazes et al. 2013). Periodic paroxysmal colics, reduced stress, intussusception, and cecal rupture conditions of the animals can be the only evident symptoms suggesting infestation (Barclay et al. 1982; Proudman and Trees 1999; Proudman and Holdstock 2000; Ryu et al. 2001).

Recent studies have reported prevalence of *A. perfoliata* in different countries, pointing to this species as the most common equine tapeworm. Its prevalence varies between 6 and 100 % (Trotz-Williams et al. 2008; Michela et al. 2009; Sangioni et al. 2009; Pavone et al. 2011; Hinney et al. 2011) In Poland, its prevalence varies from 0 to even 72 % depending on type of breeding system and geographical region (Ślivińska et al. 2009; Tomczuk 2012).

Despite the high prevalence of *A. perfoliata,* there is no best method to detect tapeworm infection in horses. Macroscopic methods of fecal analysis are rarely applied because of small size of segments and their irregular excretion. Detached proglottids are usually without eggs and morphologically do not resemble the classical segments of tapeworm (Schuster 1991; Ślivińska et al. 2009). Microscopic fecal analyses have limitations for detecting tapeworms due to a low concentration of eggs in feces (Slocombe 2006).

Coprological methods like flotation and sedimentation used routinely for the detection of carnivores’ cestodosis are often unreliable. The McMaster technique has also a very low sensitivity for anoplocephalosis (Proudman and Edwards 1992; Nilsson et al. 1995; Meana et al. 1998; Williamson et al. 1998). The most recommended coprological method is a sedimentation–flotation technique with sensitivity estimated above 50 % (Gundlach et al. 2003; Williamson et al. 1998). PCR and ELISA tests compared to standard coprological methods are more sensitive but procedures are time- and cost- consuming and also restricted to specialist laboratories (Traversa et al. 2008). What is more, relatively long and variable prepatent period of invasion impacts the diagnostics as well. High tapeworm burdens usually increase the sensitivity of coprological methods (Proudman and Edwards 1992; Williamson et al. 1998). However, previous studies have shown poor correlation between infection intensity and sensitivity of coprological methods used routinely for the detection of *A. perfoliata* (Gundlach et al. 2003; Gawor 1995). This has possible association with tapeworm maturity and the number of gravid proglottids. Therefore, the aim of this study was to prove the sensitivity of standard coproscopic methods and a modified sedimentation-flotation method on the basis of post-mortem examinations confirming the prevalence of *A. perfoliata* and the stage of tapeworms’ maturity.

**Fig. 1:**
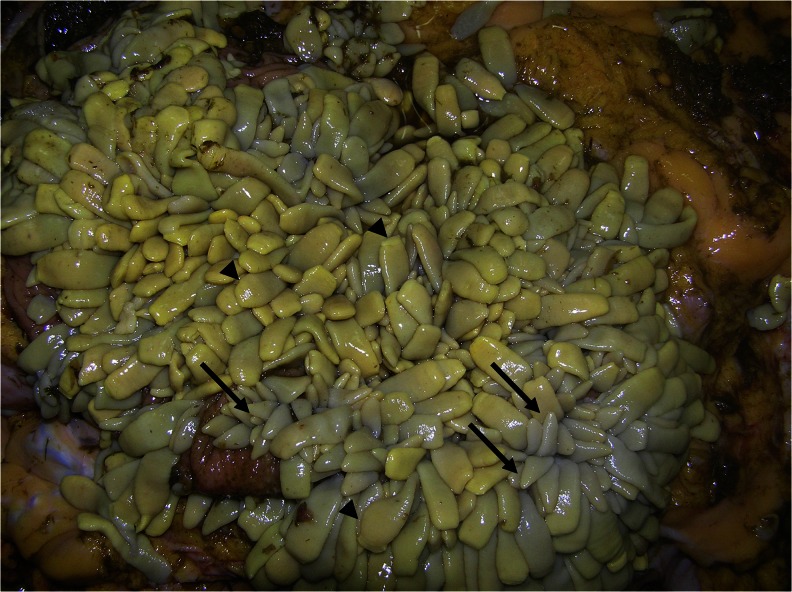
Beginning of patent period during the invasion of *Anoplocephala perfoliata* (horse number 25). Immature tapeworms (*arrows*) and tapeworms with gravid segments (*arrow heads*)

**Fig. 2:**
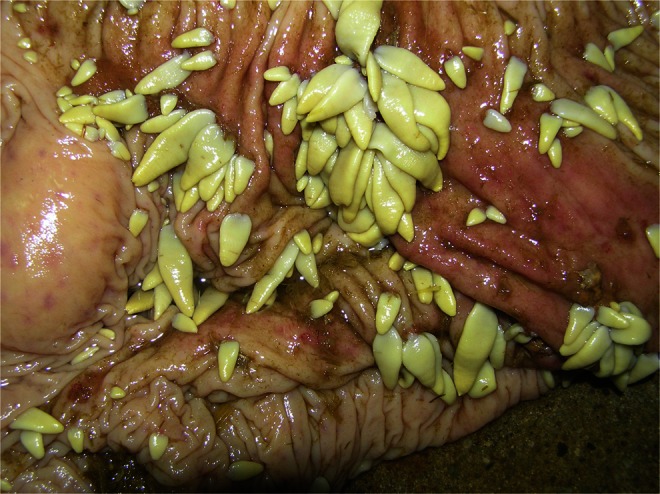
Prepatent period of invasion (horse number 13). Lance-shaped-like strobila of immature *Anoplocephala perfoliata*

## Materials and methods

Parasitological post-mortem examinations of gastrointestinal tracts of 487 slaughtered horses were performed to diagnose the intensity of tapeworm invasion between August and November 2010. Species identification and maturity stages of tapeworms were determined considering morphological characteristics and the presence of eggs in the gravid segments (Schuster 1991). For each positive horse (tapeworms present in the cecum), fecal samples were collected from the rectum or small colon for coproscopic examinations. Samples were analyzed by the conventional coproscopical methods (Gundlach et al. 2003; Schnieder 2006).

Four methods were applied: flotation (*F)* (flotation solution-saturated sodium chloride; specific gravity 1.2 g/mL), sedimentation (*Sed*), modified sedimentation–flotation (*Sed-F*), and McMaster (*McM)* technique. Fecal samples for each method weighed 5 g flotation and decantation method, 50 g modified sedimentation–flotation techniques, 2 g McMaster technique.

The modified sedimentation–flotation technique involved a 50 g fecal sample poured over with 0.0025 % Tween 80 solution in 400 mL beakers, homogenized for 60 s and allowed to stand for 30 min. The suspension was filtered through a 200-μm sieve to high-capacity centrifuge tubes. The filtrate was centrifuged at 2,600 *g* for 10 min. After supernatant removing, glass beads and a saturated solution of NaCl and sucrose (specific gravity 1.25 g/mL) were added to the precipitate. The suspension was homogenized and transferred (after filtration of bead) to a 100-mL tube. Samples were centrifuged at 2,600 *g* for 2 min. The flotation solution was supplemented to the formation of a convex meniscus. Tubes were covered with cover glasses. After a 30-min flotation, a small portion of solution was drained by a pipette, cover glasses removed gently, and examined material was placed on a slide glass.

For the quantitative test and statistical analyses in each of the applied methods, tapeworm eggs were counted for determination of fecal egg count (FEC). The number of found eggs was calculated on the whole surface of the glass slide (flotation and modified sedimentation–flotation techniques) or Petri dish (sedimentation). In the McMaster technique, eggs were counted within the engraved area of both chambers.

### Statistical analysis

Prevalence values (percentage of animals infected) are shown with 95 % confidence limits (95 % CL), the latter having been calculated according to Rohlf and Sokal (1995). All data were tested for normality. Method sensitivity (*S*) was calculated as follows:

$$ S=\frac{\mathrm{number}\kern.2em \mathrm{of}\kern.2em \mathrm{positive}\kern.2em \mathrm{samples}}{\mathrm{number}\kern.2em \mathrm{of}\kern.2em \mathrm{all}\kern.2em \mathrm{samples}}\times 100\% $$

Sensitivity was calculated within two factors. *S*_1_ was calculated using intensity of invasion (number of tapeworms) and the results of given methods. *S*_2_ was calculated using number of tapeworms with gravid proglottids and the result of given methods.

Pearson’s correlation coefficient was used to estimate the relationship between log_10_ intensity of infection and fecal egg count (FEC) and between log_10_ number of tapeworms with gravid segments and FEC. All means are reported ± standard error of mean (S.E.M) unless other stated. All statistical analyses were conducted using R version 2.12.0 (R Development Core Team, 2010) and MS Excel, 2010. A probability of <0.05 was considered significant.

## Results

In total, 36 horses were infected with *A. perfoliata* with overall prevalence of 7.4 % (4.5–11.8). No other tapeworm species were found in examined horses. The invasion intensity of *A. perfoliata* varied from 3 to 2,069 individuals (mean worm burden = 265 ± 76.1). Tapeworms with gravid segments were present in 29 horses. The intensity of invasion in relation to tapeworms with gravid segments ranged from 3 to 1,191 tapeworms (mean worm burden with gravid segments = 164 ± 54.1). Tapeworms with gravid segments constituted 62 % of all isolated individuals (Table 1). The detection of *A. perfoliata* invasion varied within applied coproscopic techniques. The number of *A. perfoliata* positive fecal samples was 3.5 times higher using the sedimentation–flotation methods than flotation (*S*_1_ = 58.3 and 16.7 %, respectively). Sedimentation and McMaster’s methods found relatively small number of positive samples (three and one positive horses, respectively). The sensitivity (*S*_2_) of the coproscopic techniques during the patent period increased significantly. Sedimentation–flotation and flotation method showed 1.24 times higher sensitivity in patent period rather than in prepatent period of infection. Sensitivity (*S*_2_) increased also for sedimentation and McMaster’s methods; however, still these methods were not highly sensitive (*S*_2_ = 10.3 and 3.4 %, respectively) (Table 2).

**Table 1:** Results of post-mortem examination and coproscopic methods of *A .perfoliata* positive horses

No. of horse	Post-mortem examination		FEC			
	Number of tapeworms (intensity of infestation)	Number of tapeworms with gravid proglottids	Sed	McM	*F*	Sed-F
1	98	69	0	0	0	8
2	35	0	0	0	0	0
3	112	14	0	0	0	0
4	43	0	0	0	0	0
5	165	148	0	0	0	12
6	38	4	0	0	0	0
7	67	53	0	0	0	7
8	32	7	0	0	0	0
9	9	9	0	0	0	3
10	12	12	0	0	0	2
11	43	7	0	0	0	0
12	27	0	0	0	0	0
13	145	4	0	0	0	0
14	66	66	1	0	0	17
15	137	73	0	0	1	15
16	78	57	0	0	0	9
17	3	0	0	0	0	0
18	912	691	0	0	2	24
19	524	486	0	0	0	18
20	747	633	2	0	2	33
21	248	32	0	0	0	0
22	595	43	0	0	0	5
23	254	32	0	0	0	4
24	65	53	0	0	0	15
25	2,069	1,172	0	0	2	31
26	1,448	1,191	2	1	4	85
27	22	0	0	0	0	0
28	162	37	0	0	0	9
29	38	0	0	0	0	0
30	1,076	830	0	0	0	19
31	52	48	0	0	0	7
32	65	65	0	0	0	25
33	68	68	0	0	3	29
34	48	15	0	0	0	0
35	9	3	0	0	0	0
36	43	0	0	0	0	0
Total	36	29	3	1	6	21

**Table 2:** Sensitivity of coproscopic methods used for *A. perfoliata* diagnosis

Technique	McM	Sed	*F*	Sed-F
	Sensitivity			
*S*_*1*_	2.8	8.3	16.7	58.3
*S*_*2*_	3.4	10.3	20.7	72.4
	Pearson’s correlation coefficient			
Intensity (number of both mature and juvenile stages of *A. perfoliata)*	0.40	0.44	0.64	0.69
*P* value	0.134	0.065	<0.0001	<0.0001
Number of tapeworms with gravid segments	0.52	0.54	0.71	0.8
*P* value	0.012	0.007	<0.0001	<0.0001

## Discussion

Diagnosing a tapeworm in humans and animals is difficult and fraught with the possibility of error. In addition, there are some differences in the diagnosis of tapeworms from two separate orders, Cyclophyllidea and Pseudophyllidea. This is caused by dissimilarities in the structure of the uterus and various manners of elimination of the invasive eggs. Tapeworms from the Pseudophyllidea order, such as *Diphyllobothrium latum*, which is common in central Europe, in humans and carnivores, may be diagnosed by classical decantation, flotation methods, or sedimentation–flotation methods using appropriate parameters of flotation fluids (high specific weight). Characteristic eggs (not invasive, with an operculum, morphologically similar to Trematoda eggs) in the mentioned tapeworm species are not stored in the gravid segments but regularly excreted in the feces during the patent period (Raether and Hänel 2003). The sensitivity of the diagnostic methods will depend on the intensity of the invasion and the degree of tapeworm maturity (Figs. 1 and 2). Regarding tapeworms from the *Cyclophyllidea* order, eggs are not regularly excreted outside the uterus but are stored inside, where they mature. After isolation and disintegration of gravid segments (what takes place in the environment), after the expulsion of the hosts, the eggs are dispersed in the feces. This process does not happen often. In some cases, the disintegration of segments occurs in the intestine and then isolated eggs will be present in the feces. In this case, application of sedimentation, flotation, or sedimentation–flotation techniques has a limited usage. Depending on the tapeworm species, only a small part of the eggs will be present in the feces. The remaining eggs will still be found in the uterus, in the gravid segments excreted. Moreover, the segments are not always excreted regularly.

**Fig. 3:**
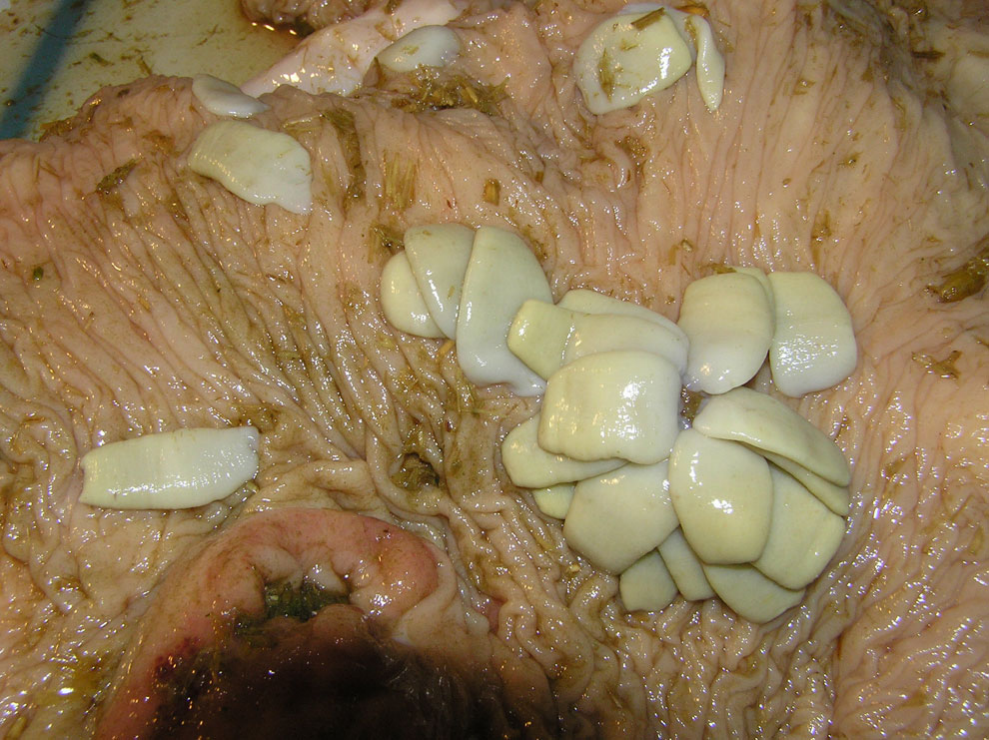
Invasion in the patent period (horse number 14). All tapeworms with gravid segments

Such an incidence occurs in invasions of *Cyclophyllidea* tapeworms (family: *Taeniidae*) in carnivores. In these invasions, the application of these methods is not sufficiently reliable. The coproscopic technique should be additionally extended by macroscopic and microscopic examinations of fecal samples for the existence of segments of tapeworms (Raether and Hänel 2003). The family *Anoplocephalidae* is an exception inside in the order *Cyclophyllidea*. Segments of these tapeworms are specifically short what helps to disintegrate and release the eggs (Schuster 1991). Frequently, eggs appear in the course of the invasion of these tapeworms in the feces of the infected animals before reaching the defragmentation of the strobila and gravid segment separation. This is particularly evident regarding the *A. perfoliata* tapeworms. A relatively small number of segments and then the damage to the walls between the segments cause eggs to disperse in the intestine of the host before the detachment of the segment. This occurrence probably has a minor significance in tapeworms achieving significant size, such as *Moniezia* spp. or *Anoplocephala magna*, where large fragments of strobila are disconnected. *A. perfoliata* is a specific species of tapeworm, in which, despite belonging to the order *Cyclophyllidea*, eggs occur regularly in the feces during the patent period (Fig. 3). It is similar with Pseudophyllidea tapeworms, although a different mechanism is at work. Taking that into consideration the diagnostics of the invasion of *A. perfoliata* tapeworms may be applied to the coproscopic method and that effectiveness will be differentiated.

Despite the progress in parasitology diagnostics and availability of serological and molecular techniques, the routine diagnostics of equine performance test is based on coproscopic examinations (Gundlach et al. 2003; Hearn and Hearn 1995; Skotarek et al. 2010; Williamson et al. 1998). This study compared the effectiveness of basic coproscopic methods. The sedimentation method is rarely used in the study of feces in horses. Sedimentation may be recommended in analysis of equine fecal samples due to the presence of *Fasciolla hepatica* in horses (Sadzikowski et al. 2009).

Small number of excreted eggs in relation to volume of horse feces makes the classic sedimentation or flotation methods useless. Our findings indicating the sensitivity at a level of 2.8–16.7 % are consistent with the other results which reported the sensitivity at a level of 2–13 % (Hearn and Hearn 1995). Numerous intensive infections demonstrated relatively a small number of eggs. However, the opposite phenomenon was also observed; invasions with a negligible intensity produced a relatively high (allowing for their detection) number of eggs in the feces. Considering the structure of infestations, it was also found that invasions with tapeworms at various maturity stages were undetectable or showed a small number of eggs in feces. It may be assumed that parasites were at an early stage of egg production, which explains a small number of eggs in the feces. However, the invasions attributed only to mature parasites (all tapeworms matured, horses no. 9, 12, 32, and 33) demonstrated a large number of eggs in the feces in relation to the intensity of the invasion. Our results showed that the sensitivity of the sedimentation–flotation methods increases by more than 14 % when we analyzed the feces only from horses in patent stage of invasion. Moreover, the Pearson’s correlation coefficient between log10 number of tapeworms with gravid segments and FEC was higher than log10 total intensity of infection and FEC.

These relationships also confirmed by others (Gasser et al. 2005; Traversa et al. 2008) may explain varying effectiveness of coproscopic techniques during the invasion of tapeworms in horses. Another important factor that determines the efficacy of the coproscopic techniques is the weight of analyzed samples, which is relatively small (2 to 3 g) in the McMaster, flotation, and sedimentation techniques. The application of the sedimentation–flotation technique allowing for a marked increase in the mass of the sample and the use of flotation fluids with a high specific gravity considerably increases the detection sensitivity up to 38–42 % (Rehbein et al. 2011; Williamson et al. 1998), 54 % (Meana et al. 1998), and 61 % (Proudman and Edwards 1992). Our findings showed a 58.3 % sensitivity confirmed the results mentioned above. The detection limit for the sedimentation–flotation techniques is 10 tapeworms at the patent invasion stage (Williamson et al. 1998). In the present study, we were able to detect nine matured tapeworms by the sedimentation–flotation method. The infection intensity and the stage of parasite maturity decide on the sensitivity of the sedimentation–flotation methods. Kjaer et al. (2007) provided evidence that the detection of *A. perfoliata* invasion increases to 89 % when the intensity of infection is higher than 20 tapeworms with uterine segments.

Recently, immunological techniques have been introduced for tapeworm diagnosis; the ELISA detects the presence of IgG antibodies in the serum or excretory–secretory antigens in the feces of the infected animals (Höglund et al. 1995; Kania and Reinemeyer 2005; Traversa et al. 2008). These methods also pose a risk for errors because the antigens are present in the serum of horses, 3 weeks after the infection and may persist at detectable levels within 28 days after the parasite elimination. Moreover, their titer and the IgG antibody levels depend on the invasion intensity (Traversa et al. 2008). Therefore, the sensitivity of serological methods is comparable to sedimentation–flotation techniques and ranges from 70–74 % (Rehbein et al. 2002). On the other hand, 97 % sensitivity of PCR-based diagnostics seem to be much more sensitive by the detection of parasite-specific DNA sequences in the feces of animals (Drogemuller et al. 2004; Schnieder 2006). Theoretically, the presence of one tapeworm cell or egg is enough to amplify a selected DNA fragment and read a positive result. Nevertheless, this technique is not always able to detect the invasion during the prepatent period. Method sensitivity depends significantly on the stage of parasite maturity which is associated with the development of parasite invasions. Because of the seasonality of infection, the maturity may peak at different times in central European climate conditions (Tomczuk 2012). Thus, the variability of effectiveness should be taken into account when the examination performed by the same technique is carried out in different seasons. Therefore, the decisions concerning antiparasitics treatment should include not only the results of examination but also the knowledge of invasion conditions and observations of nonspecific clinical symptoms.

To sum up, it may be stressed that tapeworm invasion depends on several specific factors and the sensitivity of routine coproscopic methods is inadequate. The effective detection of tapeworm infection compared to serological techniques is possible when the sedimentation–flotation methods are used.
